# Opposing roles for mammary epithelial-specific PPARγ signaling and activation during breast tumour progression

**DOI:** 10.1186/s12943-015-0347-8

**Published:** 2015-04-15

**Authors:** Anthony J Apostoli, Jennifer M Roche, Mark M Schneider, Sandip K SenGupta, Michael A Di Lena, Rachel E Rubino, Nichole T Peterson, Christopher JB Nicol

**Affiliations:** Department of Pathology and Molecular Medicine, Queen’s University, Kingston, ON Canada; Division of Cancer Biology and Genetics, Queen’s Cancer Research Institute (QCRI), Kingston, ON Canada; Department of Biomedical and Molecular Sciences, Queen’s University, Kingston, ON Canada

**Keywords:** Breast cancer, PPARγ, Mammary epithelial cells, Knockout mouse model, Chemotherapy

## Abstract

**Background:**

Among women worldwide, breast cancer is the most commonly diagnosed cancer, and the second leading cause of cancer-related deaths. Improved understanding of breast tumourigenesis may facilitate the development of more effective therapies. Peroxisome proliferator-activated receptor (PPAR)γ is a transcription factor that regulates genes involved in insulin sensitivity and adipogenesis. Previously, we showed, using 7,12-dimethylbenz [a] anthracene (DMBA)-treated haploinsufficient PPARγ mice, that PPARγ suppresses breast tumour progression; however, the PPARγ expressing cell types and mechanisms involved remain to be clarified. Here, the role of PPARγ expression and activation in mammary epithelial cells (MG) with respect to DMBA-mediated breast tumourigenesis was investigated.

**Methods:**

PPARγ MG knockout (PPARγ-MG KO) mice and their congenic, wild-type controls (PPARγ-WT) were treated once a week for six weeks by oral gavage with 1 mg DMBA dissolved in corn oil and maintained on a normal chow diet. At week 7, mice were randomly divided into those maintained on a normal chow diet (DMBA Only; PPARγ-WT: n = 25 and PPARγ-MG KO: n = 39) or those receiving a diet supplemented with the PPARγ ligand, rosiglitazone (ROSI, 4 mg/kg/day) (DMBA + ROSI; PPARγ-WT: n = 34 and PPARγ-MG KO: n = 17) for the duration of the 25-week study.

**Results:**

Compared to DMBA Only-treated PPARγ-WTs, both breast tumour susceptibility and serum levels of proinflammatory and chemotactic cytokines, namely IL-4, eotaxin, GM-CSF, IFN-γ, and MIP-1α, were decreased among PPARγ-MG KOs. Cotreatment with ROSI significantly reduced breast tumour progression among PPARγ-WTs, correlating with increased BRCA1 and decreased VEGF and COX-2 protein expression levels in breast tumours; whereas, surprisingly DMBA + ROSI-treated PPARγ-MG KOs showed increased breast tumourigenesis, correlating with activation of COX-2.

**Conclusion:**

These novel data suggest MG-specific PPARγ expression and signaling is critical during breast tumourigenesis, and may serve as a strong candidate predictive biomarker for response of breast cancer patients to the use of therapeutic strategies that include PPARγ ligands.

**Electronic supplementary material:**

The online version of this article (doi:10.1186/s12943-015-0347-8) contains supplementary material, which is available to authorized users.

## Introduction

Breast cancer is the most commonly diagnosed form of cancer among women worldwide with 1.7 million new cases identified and over 500,000 breast cancer-related deaths in 2012 [1]. Despite advances in early detection and treatment for many types of breast tumours, it remains difficult to predict which patients will suffer from aggressive forms of disease or respond poorly to current therapies. More work is needed to identify biomarkers that may reduce the number of deaths and improve quality of life for patients diagnosed with breast cancer.

Peroxisome proliferator-activated receptor (PPAR)γ is a transcription factor that is primarily expressed in adipocytes [2], as well as mammary epithelial (MG) cells [3], and a majority of human breast tumour cell lines [4,5]. It regulates the expression of genes involved in glucose and lipid metabolism, with an emerging role in breast tumourigenesis [6]. The mechanisms by which PPARγ regulates gene expression are best reviewed elsewhere [7]. Ligands for PPARγ include synthetic drugs from the thiazolidinedione (TZD) class [8]. Rosiglitazone (ROSI), a TZD family member, is a potent activator of PPARγ and prescribed to successfully treat some patients with Type II diabetes [9].

A breast tumour suppressor role for PPARγ was first demonstrated *in vitro* when treatment of human MCF-7 and MDA-MB-231 breast cancer cells with PPARγ ligands resulted in decreased cell proliferation, promotion of differentiation, and induction of apoptosis [4,5,10,11]. We provided the first direct *in vivo* evidence that PPARγ normally stops the growth and spread of breast and other tumour progression in a 7,12-dimethylbenz[a] anthracene (DMBA)-treated haploinsufficient PPARγ^(+/-)^ mouse model [12]. To better define the mammary cell-specific importance of PPARγ during breast tumourigenesis, we more recently showed that *in vivo* expression and activation of PPARγ in both virgin mammary stromal adipocytes and post-lactational secretory epithelial cells protects against DMBA-induced breast tumourigenesis [13,14]. Here we sought to explore the role of virgin mammary epithelial cell (MG)-specific PPARγ signaling and activation during DMBA-mediated breast tumourigenesis using conditional PPARγ-MG KO mice. It was hypothesized that MG-specific PPARγ expression is protective during breast tumourigenesis, and that this effect could be amplified via ROSI activation of PPARγ in MG cells. Here we unveil evidence that MG-specific PPARγ expression enhances early breast tumour events; whereas, more importantly activation of MG-specific PPARγ-dependent signaling reduces breast tumour progression.

## Results

**Figure 1:**
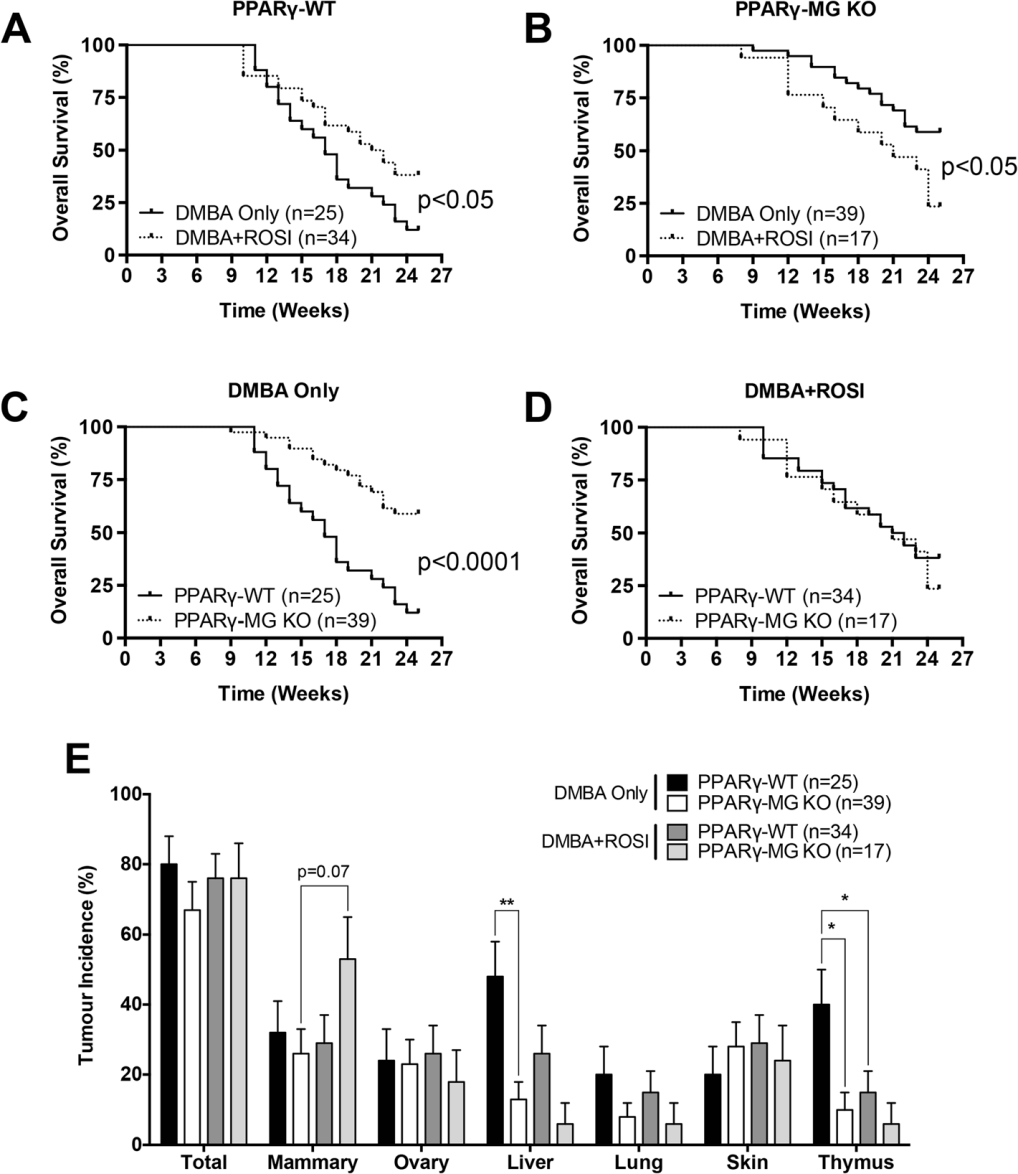
*In vivo* effects of MG-specific PPARγ loss on survival and total tumour outcomes. Overall survival outcomes for **(A)** PPARγ-WT and **(B)** PPARγ-MG KO mice are shown. Solid lines, DMBA Only treatment; broken lines, DMBA + ROSI treatment. Overall survival for **(C)** DMBA Only- and **(D)** DMBA + ROSI-treated mice are shown. Solid lines, PPARγ-WTs; broken lines, PPARγ-MG KOs. **(E)** Tumour incidences are shown for each strain across each treatment group for total, mammary, ovarian, liver, lung, skin and thymic tumours. *, p < 0.05; **, p < 0.01.

Based on observations in our lab and previous reports [15], PPARγ-WT and PPARγ-MG KO mice are not prone to spontaneous tumour formation, suggesting any tumours that arose were a result of DMBA initiation. In regards to tumourigenic response, overall survival (OS) for PPARγ-WT and PPARγ-MG KO mice are shown in Figure 1A and B respectively, and for DMBA Only-treated and DMBA + ROSI-treated mice are shown in Figure 1C and D respectively. Within genotypes, DMBA + ROSI-treated PPARγ-WTs had a significantly improved OS compared to their respective DMBA Only-treated controls (respective median OS: 21.5 vs. 17 weeks, p < 0.05). Interestingly, among PPARγ-MG KO mice, cotreatment significantly worsened OS outcomes compared to DMBA Only-treated controls (respective median OS: 21 weeks vs. undefined, p < 0.05). Among DMBA Only-treated groups, PPARγ-MG KO mice showed a strong statistically significant advantage in OS compared to PPARγ-WTs (p < 0.0001); however, this difference was not retained between DMBA + ROSI-treated genotypes.

Tumours were differentially observed in tissues among all groups, and were consistent with the pattern of DMBA-initiated tumourigenesis (Table 1). In the DMBA Only-treated group, PPARγ-WT mice had a total tumour incidence of 80 ± 8% compared to 67 ± 8% for PPARγ-MG KOs (Figure 1E). In the DMBA + ROSI group, total tumour incidence was similar for PPARγ-WTs (76 ± 7%) and PPARγ-MG KO (76 ± 10%) mice. In DMBA Only-treated mice, mammary tumour incidences were modestly higher among PPARγ-WTs (32 ± 9%) compared to PPARγ-MG KOs (26 ± 7%). In contrast, between DMBA + ROSI-treated strains, PPARγ-MG KO mice had a ~2-fold higher mammary tumour incidence compared to PPARγ-WTs (53 ± 12 vs. 29 ± 8%, respectively), although this trend was not statistically significant. Further, DMBA + ROSI-treated PPARγ-MG KO mice exhibited a ~2-fold higher incidence of mammary tumours compared to DMBA Only-treated PPARγ-MG KOs (53 ± 12% vs. 26 ± 7%, respectively) in a trend that approached statistical significance (p = 0.07).

**Table 1:** **DMBA-induced tumours in PPARγ-WT and PPARγ-MG KO mice**

	**DMBA Only-treated mice**		**DMBA + ROSI-treated mice**	
	**PPARγ-WT (n = 25)**	**PPARγ-MG KO (n = 39)**	**PPARγ-WT (n = 34)**	**PPARγ-MG KO (n = 17)**
***Mammary tumour type***		***Tumours/Mouse (# Tumours)***		
**Benign tumour**	**0.20 (5)**	**0.26 (10)**	**0.15 (5)**	**0.18 (3)**
Squamous cyst	0.12 (3)	0.08 (3)	0.09 (3)	0.06 (1)
Spindle tumour	0.04 (1)	−	−	−
Adenoma	0.04 (1)	−	0.06 (2)	−
Lipoma	−	0.03 (1)	−	−
Other	−	0.15 (6)	−	0.12 (2)
**Squamous cell carcinoma**	**0.08 (2)**	**0.03 (1)**	**0.26 (9)**	**0.29 (5)**
**Spindle cell carcinoma**	**0.08 (2)**	**−**	**−**	**−**
**Adenocarcinoma**	**−**	**−**	**0.12 (4)**	**−**
**Other carcinoma**	**0.08 (2)**	**0.03 (1)**	**−**	**0.29 (5)**
***Total mammary tumours***	**0.44 (11)**	**0.31 (12)**	**0.53 (18)**	**0.76 (13)**
**Benign mammary**	**0.20 (5)**	**0.26 (10)**	**0.15 (5)**	**0.18 (3)**
**Malignant mammary**	**0.24 (6)**	**0.05 (2)**	**0.38 (13)**	**0.59 (10)**
***Non-mammary tumour/tissue affected***		***Tumours*** **/** ***Mouse (# Tumours)***		
**Skin**	**0.20 (5)**	**0.41 (16)**	**0.35 (12)**	**0.41 (7)**
**Ovarian/Uterine**	**0.24 (6)**	**0.26 (10)**	**0.26 (9)**	**0.13 (3)**
**Thymus**	**0.40 (10)**	**0.10 (4)**	**0.15 (5)**	**0.06 (1)**
**Spleen**	**0.04 (1)**	**0.03 (1)**	**−**	**−**
**Liver**	**0.48 (12)**	**0.13 (5)**	**0.26 (9)**	**0.06 (1)**
**Lung**	**0.20 (5)**	**0.08 (3)**	**0.15 (5)**	**0.06 (1)**
**Gastrointestinal**	**0.04 (1)**	**0.05 (2)**	**0.06 (2)**	**0.06 (1)**
**Lymphoma**	**−**	**0.13 (4)**	**0.03 (1)**	**−**
***Total tumours***	**2.04 (51)**	**1.46 (57)**	**1.79 (61)**	**1.59 (27)**
**Benign total**	**0.64 (16)**	**0.82 (32)**	**0.74 (25)**	**1.06 (18)**
**Malignant total**	**1.40 (35)**	**0.64 (25)**	**1.05 (36)**	**0.53 (9)**

The number of breast tumours per mouse (multiplicity) is indicated with the total number in parenthesis. Mammary tumours were also sub-stratified and expressed as multiplicity of benign, malignant, and metastatic tumours per genotype and treatment. Examples of benign mammary tumour subtypes are also indicated. For non-mammary tissue, the numbers of each tumour per mouse is also indicated with the total number in parenthesis. Finally, total tumours were sub-stratified and expressed as the multiplicity of benign, malignant, and metastatic tumours per genotype and treatment.

In DMBA Only-treated mice, PPARγ-MG KOs also had a significant ~3.5-fold reduction in liver tumour incidence compared to PPARγ-WTs (13 ± 5% vs. 48 ± 10%, respectively; p < 0.01). Cotreatment with DMBA + ROSI halved liver tumour incidences in both genotypes, although these changes were not significantly different. Furthermore, PPARγ-MG KOs had a significant ~4-fold decrease in thymic tumour incidence compared to PPARγ-WT mice in the DMBA Only-treated group (10 ± 5% vs. 40 ± 10%, respectively; p < 0.05). Among cotreated groups, thymic tumour incidences were not different between genotypes but did significantly decrease by ~3-fold among PPARγ-WT mice compared to their respective DMBA Only-treated PPARγ-WT controls (p < 0.05).

**Figure 2:**
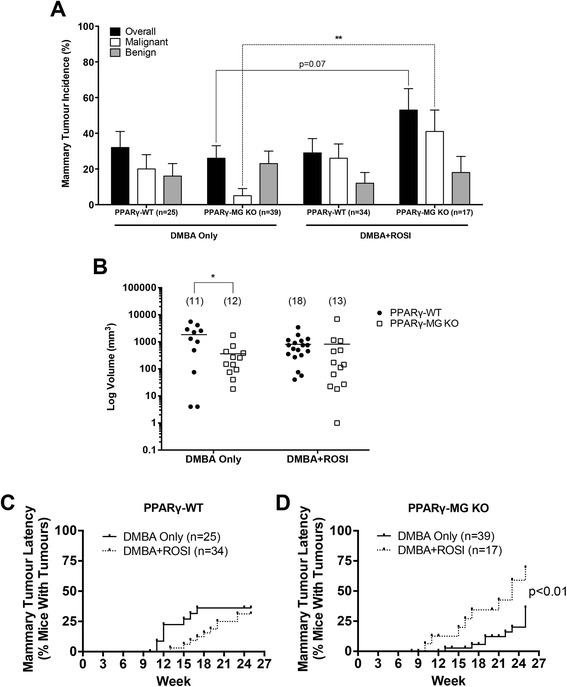
*In vivo* effects of MG-specific PPARγ deletion on mammary tumour incidence, tumour volume and latency. **(A)** Mammary tumour incidences, as well as incidences of benign and malignant mammary tumours, are shown for each strain across each treatment group. **, p < 0.01. **(B)** Mammary tumour volumes were calculated using the standard formula (L × W^2^/2) and are expressed as mm^3^ on a log scale. Solid lines, mean tumour volume for each strain; solid circles, PPARγ-WTs; open squares, PPARγ-MG KOs; *, p < 0.05. Mammary tumour latency is expressed as the percentage of palpable mammary tumours within **(C)** PPARγ-WT and **(D)** PPARγ-MG KO strains in a given week. Solid lines, DMBA Only treatment; broken lines, DMBA + ROSI treatment.

When mammary tumours were analyzed by pathological stage (Figure 2A), DMBA Only-treated PPARγ-MG KO mice exhibited a reduction in malignant mammary tumours versus PPARγ-WTs (5 ± 4% vs. 20 ± 8%, respectively; not significant). DMBA + ROSI cotreatment did not significantly change PPARγ-WT malignant mammary tumour incidence, but intriguingly, significantly increased malignant mammary tumour incidence by ~8-fold in PPARγ-MG KO mice (p < 0.01). For either genotype treated with DMBA only versus DMBA + ROSI, the incidences of benign mammary tumours were non-significantly reduced in PPARγ-WTs (16 ± 7% vs. 12 ± 6% respectively) and PPARγ-MG KOs (23 ± 7% vs. 18 ± 9%, respectively).

Mammary tumours were measured (length and width) to monitor volumes as soon as they became palpable (Figure 2B) [16]. DMBA Only-treated PPARγ-MG KO mice had a significant ~5-fold decrease in mean mammary tumour volume compared to similarly treated PPARγ-WTs (mean log volume: 360.6 mm^3^ vs. 1843 mm^3^, respectively; p < 0.05). Cotreatment with DMBA + ROSI abolished this genotypic difference, and resulted in similar mean mammary tumour volumes via increases in PPARγ-WTs (806.9 mm^3^) and decreases in PPARγ-MG KOs (818.0 mm^3^). The effects of treatment on mammary tumour volumes within each genotype were not statistically significant.

Among PPARγ-WT mice, palpable mammary tumours were first observed following DMBA treatment at week 11, and at week 13 in the DMBA + ROSI-treated group (Figure 2C). With respect to mammary tumour latency, 25% of DMBA Only-treated PPARγ-WTs developed palpable tumours by week 15, whereas this trended toward week 21.5 in DMBA + ROSI-treated PPARγ-WT mice. Interestingly, DMBA Only-treated PPARγ-MG KOs first developed palpable tumours by week 13, in comparison to DMBA + ROSI-treated PPARγ-MG KOs in which palpable tumours were noted as early as week 10. Twenty-five percent of DMBA Only-treated PPARγ-MG KO mice developed palpable mammary tumours by week 25, and this significantly declined to week 16 in DMBA + ROSI-treated PPARγ-MG KOs (p < 0.01). Similarly, DMBA + ROSI-treated PPARγ-MG KOs showed a significant decrease in mammary tumour latency compared to similarly treated PPARγ-WT mice (with 25% of mice developing palpable mammary tumours at week 16 vs. 21.5, respectively; p < 0.05).

**Figure 3:**
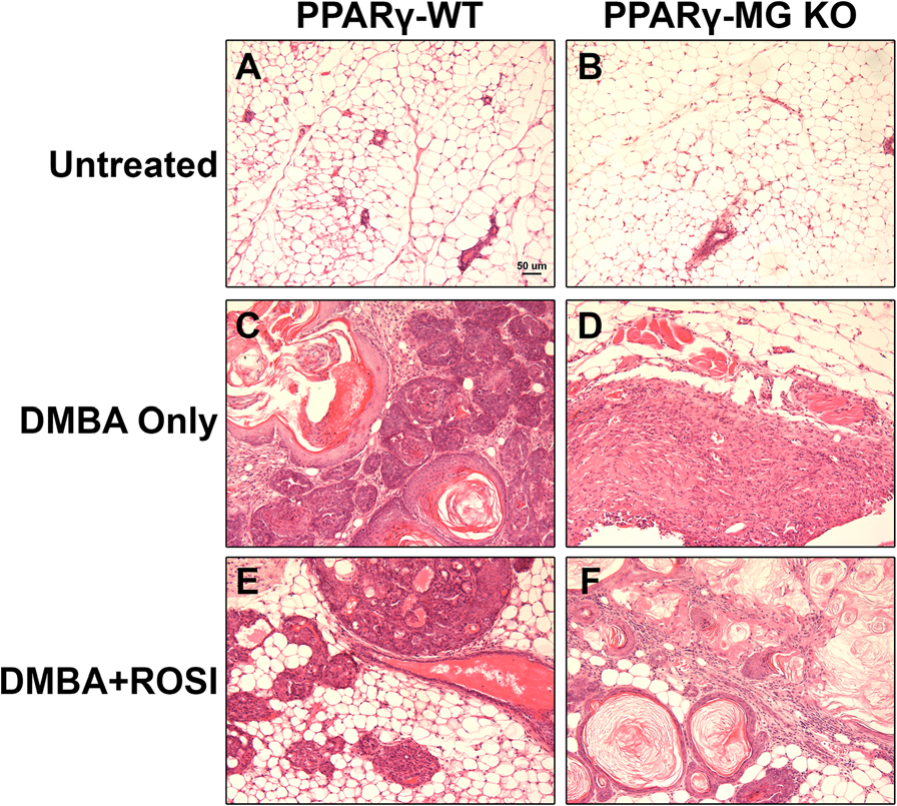
Pathological effect of MG cell-specific PPARγ deficiency on DMBA-induced mammary tumours. Mice were treated as described in the Methods section. Representative sections are shown. **(A)**, untreated PPARγ-WT mammary gland; **(B)**, untreated PPARγ-MG KO mammary gland; **(C)**, DMBA Only-treated PPARγ-WT mammary tumour; **(D)**, DMBA Only-treated PPARγ-MG KO mammary tumour; **(E)**, DMBA + ROSI-treated PPARγ-WT mammary tumour; **(F)**, DMBA + ROSI-treated PPARγ-MG KO mammary tumour. All photos taken at × 200. Scale bar, 50 μm.

Representative sections of normal mammary tissue and mammary tumours from PPARγ-WT and PPARγ-MG KO mice in each treatment group were hematoxylin and eosin (H&E) stained and examined in a blinded fashion by collaborating pathologists for changes in morphological characteristics. Untreated mammary glands collected at week 12 from either strain were not morphologically different from one another and exhibited characteristic features of normally developed mammary glands (Figure 3A and B). Both were comprised primarily of adipocytes, as expected in the mouse mammary gland. Tumours taken from PPARγ-WT mice treated with DMBA Only were primarily classified as malignant carcinomas with mixed squamous differentiation (Figure 3C). DMBA Only-treated PPARγ-MG KO mammary tumours showed comparatively more benign characteristics (Figure 3D). In DMBA + ROSI-treated mice, mammary tumours isolated from PPARγ-WT mice were primarily identified as squamous cell carcinomas (Figure 3E); whereas, those from PPARγ-MG KO mice were classified as more malignant lesions that ranged from well-to-moderately differentiated (Figure 3F).

**Figure 4:**
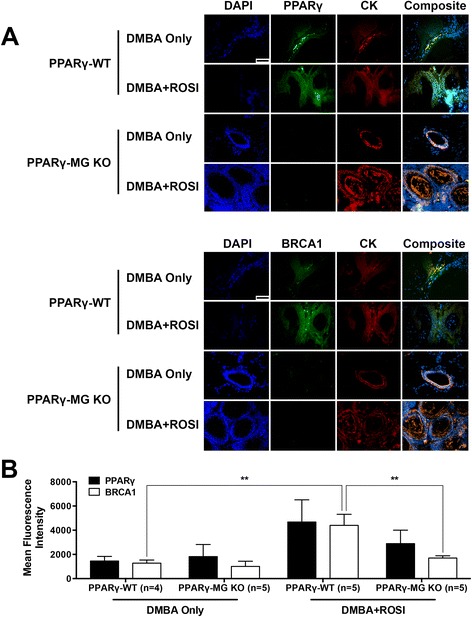
PPARγ and BRCA1 expression in DMBA-mediated mammary-derived tumours. **(A)** Representative immunofluorescence images illustrating expression of cell nuclei (DAPI; in blue), PPARγ or BRCA1 (in green) and cytokeratin (CK; in red), with an accompanying composite image, in mammary-derived tumours from PPARγ-WT and PPARγ-MG KO mice. All photos taken at × 600. Scale bar, 50 μm. **(B)** Quantification of global mean fluorescence intensity for PPARγ or BRCA1 in tumours was performed using Image Pro Plus software. **, p < 0.01.

To evaluate protein expression changes *in situ*, mean fluorescence intensities of target proteins were quantified in three regions within each analyzed mammary tumour (Figure 4A). BRCA1 was evaluated in this manner because it is a known tumour suppressor gene, whose gene promoter contains a PPRE [17]. Mammary glands from untreated strains, included for reference (Additional file 1: Figure S1), illustrate decreased PPARγ and BRCA1 expression in cytokeratin-positive MG cells in PPARγ-MG KO mice compared to PPARγ-WTs. Results show no differences in both PPARγ and BRCA1 among mammary-derived tumours from DMBA-treated PPARγ-MG KO and PPARγ-WT mice (PPARγ: 1822 ± 999 vs. 1459 ± 377, respectively and BRCA1: 1007 ± 432 vs. 1280 ± 258, respectively) (Figure 4B). Compared to DMBA Only-treated controls, irrespective of genotype, mammary tumours from mice treated with DMBA + ROSI trended toward increased PPARγ expression accompanied by increased BRCA1 expression (p = 0.09). Importantly, DMBA + ROSI treatment significantly increased BRCA1 expression ~3.5-fold in PPARγ-WT mice compared to both DMBA Only-treated PPARγ-WTs (4400 ± 915 vs. 1280 ± 258, respectively; p < 0.01), and DMBA + ROSI-treated PPARγ-MG KOs (1707 ± 180; p < 0.01).

**Figure 5:**
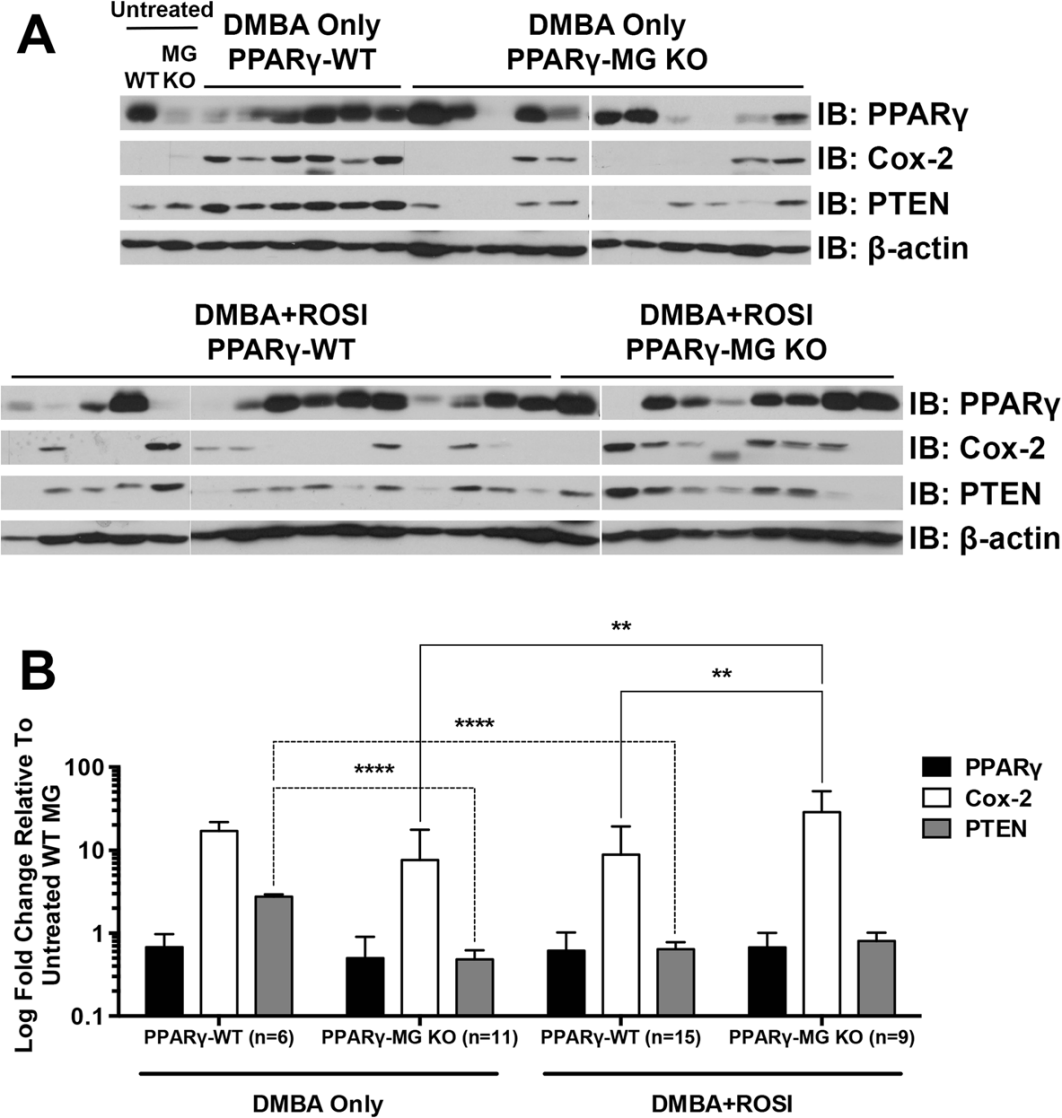
Molecular analysis from untreated mammary glands and DMBA-induced mammary tumours. **(A)** Representative protein expression changes within untreated mammary glands (MG) and *in vivo* generated mammary tumours in DMBA Only- and DMBA + ROSI-treated groups were analyzed by Western Blot as described in the Methods section. PPARγ, Cox-2 and PTEN protein levels were analyzed in untreated virgin MG from PPARγ-WT (WT) and PPARγ-MG KO (MG KO) mice, as well as all available breast tumour subtypes from both strains of mice. β-actin served as loading control. **(B)** Densitometry for PPARγ, Cox-2 and PTEN were performed on all mammary tumours using ImageJ software, and expressed as mean ± SD. Fold changes are relative to mammary tissue from untreated PPARγ-WT. Black bars, PPARγ expression; white bars, Cox-2 expression; grey bars, PTEN expression; **, p < 0.01; ****, p < 0.0001.

Protein expression changes were determined by immunoblotting in mammary tumours from DMBA Only- and DMBA + ROSI-treated strains (Figure 5A). Untreated mammary tissues from PPARγ-WT and PPARγ-MG KO mice illustrate PPARγ is reduced in the latter and are representative of results from multiple independent experiments. Densitometric analyses of protein expression within mammary tumours revealed surprisingly similar PPARγ protein levels irrespective of genotype or treatment (Figure 5B). Intriguingly, DMBA + ROSI-treated PPARγ-MG KO mammary tumours exhibited a significant ~4-fold increase in Cox-2 compared to DMBA Only-treated PPARγ-MG KOs (p < 0.01), as well as a significant ~3-fold increase in Cox-2 compared to DMBA + ROSI-treated PPARγ-WT mice (p < 0.01). A significant ~6-fold reduction in PTEN was observed among DMBA Only-treated PPARγ-MG KOs compared to similarly treated control mice (p < 0.0001). No change in PTEN expression was observed among DMBA Only- and DMBA + ROSI-treated PPARγ-MG KOs; however, it was interesting to note that ROSI cotreatment produced a significant ~4-fold reduction in PTEN in PPARγ-WT mice compared to DMBA Only-treated controls (p < 0.0001).

**Figure 6:**
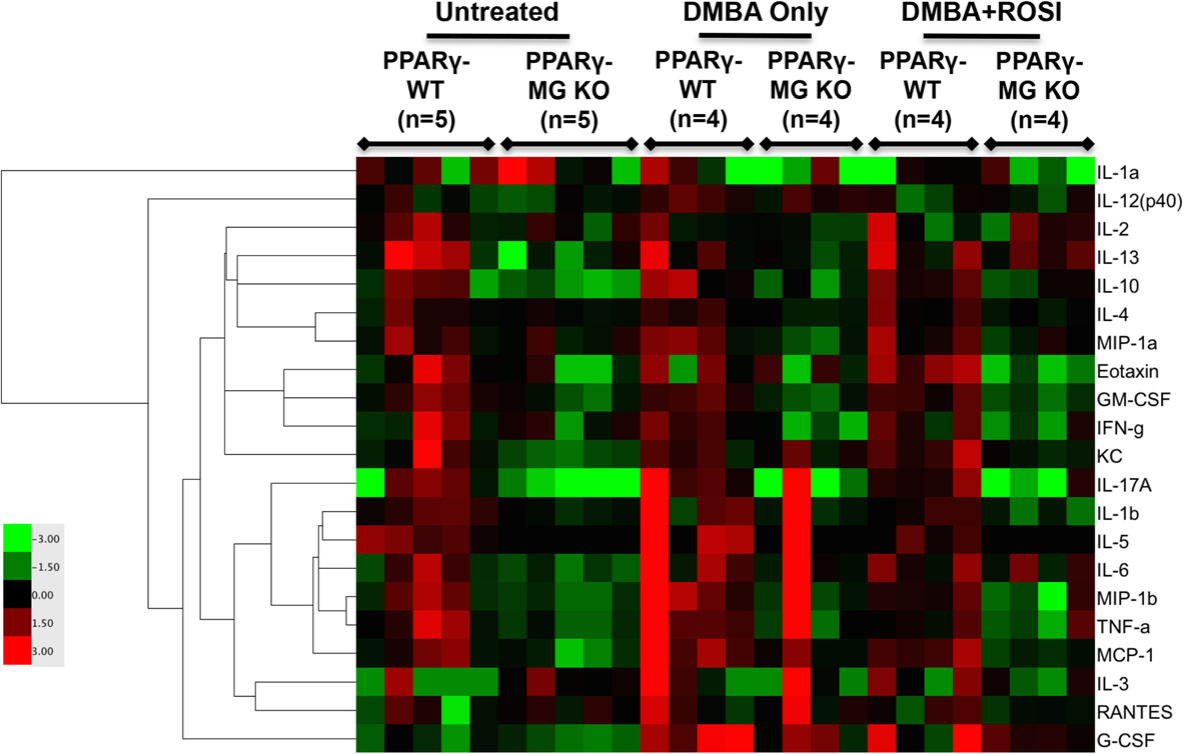
Heatmap reconstruction of 23-plex cytokine analyses resulting from MG-specific PPARγ loss. A heatmap generated from a 23-plex cytokine array illustrating serum concentrations of cytokines from untreated, DMBA Only- and DMBA + ROSI-treated PPARγ-WT and PPARγ-MG KO strains. Mean cytokine concentrations (pg/ml) are visually represented on a log scale with red, black and green indicating high, median and low, respectively (refer to colour bar). IL-9 and IL-12(p70) were omitted from the table since values were below the level of detection.

A 23-plex cytokine array was performed on serum samples from both PPARγ-WT and PPARγ-MG KO strains for untreated, DMBA Only-treated, and DMBA + ROSI-treated mice (Figure 6). Among untreated mice, there were significantly lower levels of GM-CSF (~2.5-fold; p < 0.05) observed in PPARγ-MG KOs compared to PPARγ-WT mice (Table 2). DMBA + ROSI treatment significantly reduced serum GM-CSF (~3.5-fold; p < 0.01), and non-significantly decreased serum eotaxin (~11-fold; p < 0.10), in PPARγ-MG KO mice compared to similarly treated PPARγ-WTs. Interestingly, PPARγ-MG KOs showed significantly lower levels of serum IL-4 (p < 0.01), IL-10 (p < 0.001), IL-13 (p < 0.05), eotaxin (p < 0.01), GM-CSF (p < 0.0001), IFN-γ (p < 0.05) and MIP-1α (p < 0.01), as well as a trend toward reduced levels of KC (p = 0.08), compared to PPARγ-WTs.

**Table 2:** **Serum concentrations of cytokines from untreated, DMBA Only- and DMBA + ROSI-treated strains**

	**PPARγ-WT**			**PPARγ-MG KO**		
	**Untreated (n = 5)**	**DMBA Only (n = 4)**	**DMBA + ROSI (n = 4)**	**Untreated (n = 5)**	**DMBA Only (n = 4)**	**DMBA + ROSI (n = 4)**
**Cytokine** *[signif]*	***mean ± SD; all values expressed as pg*** **/** ***ml***					
**IL-1α**	90.6 ± 56.6	93.9 ± 106.3	48.9 ± 32.9	177.0 ± 206.4	39.8 ± 66.1	36.8 ± 46.0
**IL-1β**	372.6 ± 105.3	3016.0 ± 5279.0	309.0 ± 74.0	195.4 ± 25.9	870.9 ± 1383.0	116.7 ± 91.1
**IL-2**	62.8 ± 47.0	42.4 ± 27.9	64.6 ± 80.3	34.9 ± 15.0	24.8 ± 6.1	41.8 ± 32.9
**IL-3**	6.8 ± 15.2	63.4 ± 111.5	16.0 ± 13.6	12.9 ± 6.6	25.4 ± 40.9	6.8 ± 5.6
**IL-4** *[gg]*	16.1 ± 7.9	16.4 ± 3.2	20.1 ± 10.0	11.8 ± 1.5	10.4 ± 1.2	11.0 ± 1.1
**IL-5**	40.0 ± 16.3	280.7 ± 454.0	23.2 ± 17.2	ND	77.4 ± 154.8	4.6 ± 9.3
**IL-6**	18.0 ± 16.4	215.5 ± 387.5	20.7 ± 12.0	5.7 ± 1.5	62.7 ± 106.6	14.5 ± 8.5
**IL-9**	ND	ND	ND	ND	ND	ND
**IL-10** *[ggg]*	97.3 ± 64.6	163.2 ± 114.2	117.6 ± 48.8	23.5 ± 9.5	39.7 ± 19.9	52.0 ± 20.9
**IL-12(p40)** *[tt]*	694.9 ± 299.5	1212.0 ± 302.4	625.3 ± 314.0	554.5 ± 176.1	971.7 ± 306.8	665.1 ± 227.3
**IL-12(p70)**	ND	ND	ND	ND	ND	ND
**IL-13** *[g]*	654.1 ± 638.6	410.6 ± 437.3	440.6 ± 380.4	95.4 ± 71.7	124.9 ± 33.2	248.0 ± 91.7
**IL-17A**	32.3 ± 23.8	588.5 ± 1114.0	34.9 ± 19.5	2.1 ± 3.2	150.3 ± 294.5	7.8 ± 12.6
**Eotaxin** *[gg]*	1552.0 ± 1593.0	1141.0 ± 890.4	2016.0 ± 743.7	400.4 ± 397.5	620.8 ± 482.6	188.4 ± 163.4
**G-CSF**	199.6 ± 85.9	1631.0 ± 929.1	3393.0 ± 5274.0	149.1 ± 46.6	564.3 ± 375.5	433.7 ± 121.9
**GM-CSF** *[gggg]*	420.2 ± 218.5	384.4 ± 86.8	375.3 ± 94.4	173.0 ± 68.1 *	148.6 ± 47.4	110.1 ± 75.6 Δ
**IFN-γ** *[g]*	35.4 ± 38.6	26.6 ± 10.5	23.9 ± 11.3	15.3 ± 6.8	6.8 ± 6.4	9.6 ± 7.0
**KC**	46.4 ± 62.2	25.3 ± 9.4	41.1 ± 28.2	8.8 ± 1.6	23.1 ± 11.5	14.9 ± 2.4
**MCP-1**	218.2 ± 132.6	1062.0 ± 1493.0	286.4 ± 156.2	75.8 ± 38.7	190.7 ± 132.6	95.9 ± 17.7
**MIP-1α** *[gg]*	67.2 ± 47.2	87.8 ± 38.9	82.5 ± 54.7	45.1 ± 15.2	26.3 ± 8.9	37.1 ± 11.4
**MIP-1β**	51.9 ± 36.9	525.1 ± 909.0	39.4 ± 13.2	15.2 ± 4.0	185.8 ± 335.4	17.4 ± 15.8
**RANTES**	31.5 ± 23.1	71.6 ± 76.2	40.7 ± 19.4	37.1 ± 8.5	82.1 ± 97.6	27.2 ± 3.9
**TNF-α**	1128.0 ± 992.2	19244.0 ± 36869.0	538.0 ± 207.5	272.8 ± 81.9	5763.0 ± 10948.0	363.3 ± 352.8
**VEGF**	144.6 ± 32.8 (7)	489.7 ± 420.8 (3) **	132.8 ± 33.7 (4) #	254.4 ± 57.0 (8)	167.5 ± 40.9 (4) #	137.3 ± 22.4 (4)
**Leptin**	22290.0 ± 11260.0 (7)	8040.0 ± 1968.0 (3)	25820.0 ± 18020.0 (4)	13680.0 ± 1899.9 (8)	32140.0 ± 40310.0 (4)	39560.0 ± 44040.0 (4)
**PGE Metabolites**	560.1 ± 676.7 (5)	76.7 ± 13.7 (4)	93.9 ± 51.6 (4)	141.3 ± 122.2 (4)	335.0 ± 158.5 (3)	229.0 ± 202.7 (3)

Concentrations reported as mean ± standard deviation (SD) and expressed as pg/ml. Except for VEGF, leptin, and PGE metabolites, which were analyzed with separate ELISA kits, all cytokine concentrations were obtained by a multiplex array. *, significantly different from Untreated PPARγ-WT, p < 0.05; **, significantly different from Untreated PPARγ-WT, p < 0.01; #, significantly different from DMBA Only-treated PPARγ-WT, p < 0.05; Δ, significantly different from DMBA + ROSI-treated PPARγ-WT, p < 0.05. g, genotype different, p < 0.05; gg, genotype different, p < 0.01; ggg, genotype different, p < 0.001; gggg, genotype different, p < 0.0001; tt, treatment different p < 0.01; ND, not detectable.

Given their putative relevance to mammary tumour growth, serum VEGF, leptin and PGE metabolites were also quantified by separate ELISA experiments in both untreated strains and those treated with DMBA alone or DMBA + ROSI (Table 2). No significant differences were observed in serum leptin and PGE metabolite levels between genotypes or treatment groups. In contrast, DMBA Only-treated PPARγ-MG KOs had significantly ~3-fold lower serum VEGF levels compared to similarly treated PPARγ-WTs (p < 0.05). VEGF expression was also significantly reduced ~4-fold in DMBA + ROSI compared to DMBA Only levels in PPARγ-WTs (p < 0.05), but not PPARγ-MG KOs.

## Discussion

Given recent evidence implicating a protective role for PPARγ in breast cancer [12,13,14], the MG cell-specific contribution of this receptor was evaluated during DMBA-induced breast tumourigenesis using PPARγ-MG KO and PPARγ-WT mice. Cotreatment with a gold standard PPARγ activator, ROSI, further provided the ability to identify PPARγ-dependent anti-breast tumour progression signaling pathways specific to MG cells. Other groups have examined the MG-specific contribution of PPARγ in breast cancer, using overexpression [18] and dominant negative knockout [19] approaches that only target the PPARγ1 isoform. Here, the *Cre*-loxP system was used to delete expression of both PPARγ protein isoforms, and thus, eliminate any confounding compensatory effects. In addition, the ROSI dose and regimen used here was previously shown to effectively activate PPARγ signaling [20-22] and achieve serum glucose profiles within human therapeutic ranges in mice [23,24]. Surprisingly, it was discovered that PPARγ-MG KO mice are protected more so than PPARγ-WTs during DMBA-mediated breast tumourigenesis; whereas, PPARγ activation by ROSI rescues PPARγ-WTs but renders PPARγ-MG KOs more susceptible to breast tumour progression. These findings suggest that PPARγ expression within MG cells may be a strong candidate biomarker for identifying patient populations with aggressive breast tumours, as well as aid in predicting patients likely to benefit from novel chemotherapeutic use of PPARγ activating drugs.

The findings that PPARγ-MG KO mice respond more favourably, for example in OS, than PPARγ-WTs following tumourigenic initiation by DMBA, but do worse following cotreatment with a PPARγ activating ligand were unexpected. These surprising outcomes may be explained, at least in part, by the increased total mammary tumour and malignant mammary tumour incidences, and decreased mammary tumour latency, that were observed in DMBA + ROSI-treated PPARγ-MG KOs compared to those treated with DMBA alone. Collectively, these findings suggest that PPARγ expression in MG cells and ROSI activation in mice lacking MG-specific PPARγ is potentially harmful during chemical-mediated breast tumour progression. Given that ROSI activation produced detrimental effects exclusive to knockout mice suggests that PPARγ-independent effects of this drug may be partly responsible [25]. These interesting observations underscore the importance of personalized medicine, and the need for characterizing normal breast and mammary tumour epithelial expression of PPARγ before considering TZD-like drugs as chemotherapeutic strategies for breast cancer patients. ROSI may still represent a viable chemotherapeutic option if expression of MG cell-specific PPARγ remains intact.

With respect to the mouse mammary tumour outcomes observed in these studies, both MG-specific PPARγ deficiency and activation produced similar results. Although paradoxical, these comparable outcomes may reflect similar signaling pathways resulting from cofactor mobility. For example, in the PPARγ-MG KO model, coactivators and/or corepressors normally bound by the PPARγ/RXRα complex may be released to interact with their downstream signaling targets and exert their intended effects similar to when PPARγ is activated. This may partially explain why DMBA Only-treated PPARγ-MG KOs have better OS compared to DMBA Only-treated PPARγ-WTs, but comparable to PPARγ-WTs treated with DMBA + ROSI. A similar mechanism has been reported in a PPARβ KO mouse model, whereby an antiinflammatory corepressor (Bcl-6) is free to exert its effects in both PPARβ-deficient and PPARβ-activated cell contexts [26]. RNAseq and ChIPseq assays evaluating global PPARγ/RXRα interactions with specific cofactors and gene targets would help clarify if this mechanism is involved in the context of breast tumourigenesis, but is beyond the scope of these studies.

MG-specific expression of PPARγ and BRCA1 were confirmed in untreated PPARγ-WT but abolished in PPARγ-MG KO mammary glands. Importantly, ROSI cotreatment increased PPARγ expression in mammary-derived lymphomas and carcinomas from PPARγ-WT and PPARγ-MG KO mice, but only specifically augmented BRCA1 in PPARγ-WTs. BRCA1 is a critical tumour suppressor gene that possesses a PPRE within its promoter region [17]. We have previously demonstrated that BRCA1 expression can be upregulated in fat cells via adipocyte-specific PPARγ activation [13]. Accordingly, the current study provides similar evidence that BRCA1 is a target of PPARγ in MG cells. This specific interaction may contribute to the improved outcomes observed among DMBA + ROSI-treated PPARγ-WT mice, via BRCA1-mediated DNA damage repair and/or blocking aromatase-dependent estrogen production [27].

Cox-2 is a key PG-synthesizing enzyme and a breast cancer prognostic marker of poor outcome [28]. Consequently, Cox-2 protein expression is observed in many epithelial tumours, including breast cancer [29], with increasing levels associated with advanced tumour grade [30,31]. ROSI cotreatment repressed Cox-2 in PPARγ-WT tumours, but dramatically amplified it in PPARγ-MG KOs. This marked increase in Cox-2 protein levels among DMBA + ROSI-treated PPARγ-MG KO tumours may partially explain the poor survival and mammary tumour outcomes within this study group. Indeed, Cox-2 promotes aromatase transcription [32] and renders cells resistant to apoptosis and even chemotherapy [31]; however, some of these properties may be mediated by PG levels. That we did not observe any significant differences with respect to serum PGE levels in any group provides evidence that other PG products, or perhaps even Cox-2 activity independent of PG production, may be involved in this setting and requires further study.

Although the Cox-2 gene contains a PPRE within its promoter [33,34], PPARγ-dependent and PPARγ-independent mechanisms both positively and negatively regulate Cox-2 gene transcription depending on cell- and stimulus-specific contexts [33,35,36,37]. Given Cox-2 expression was lower in DMBA + ROSI-treated PPARγ-WT tumours suggests MG cell-specific PPARγ activation may play a role in suppressing Cox-2 protein levels, which is similar to our findings with respect to mammary secretory epithelial-PPARγ [14]. On the other hand, DMBA + ROSI-treated PPARγ-MG KO tumours showed a dramatic increase in Cox-2 protein levels suggesting that a PPARγ-independent process is likely responsible. It has been demonstrated that PPARγ ligands activate Cox-2 transcription via receptor-independent stimulation of the MAPK-NF-κB pathway [38,39]. Moreover, PPARγ-independent activation of the glucocorticoid receptor by ROSI may also be responsible for increased Cox-2 gene expression [40,41], although this remain to be proven.

The PTEN gene promoter also reportedly contains a PPRE [33,34], and so it was not surprising that PTEN protein levels were markedly reduced among PPARγ-MG KO mammary tumours in the DMBA Only group. Interestingly, DMBA + ROSI-treated PPARγ-WT mice had reduced PTEN expression among mammary tumours than observed in mammary tumours from respective DMBA Only controls. This may be reflective of the decreased mammary tumour progression in DMBA + ROSI-treated PPARγ-WT mice. Taken together, these data suggest PPARγ is required for normal PTEN expression in malignant mammary tumours, but PTEN is not an early PPARγ downstream signaling target in benign mammary tumours, and may be a fruitful area for research in future studies.

Variable PPARγ protein levels were observed among mammary tumours from PPARγ-WT and PPARγ-MG KO by Western blot analysis. Among PPARγ-WT tumours, the variable pattern may reflect alternative pathways acquired during tumourigenic progression of initiated cells, some of which may silence PPARγ expression. There are indeed reports that PPARγ levels decline as human breast tissue becomes increasingly malignant [42], which is consistent with our hypothesis of its role as a suppressor of breast tumour progression. Alternatively, the inherent cellular heterogeneity of these mammary tumours, that likely contain differing amounts of PPARγ expressing stromal adipocytes, endothelial cells and immune cells, may contribute to the observed variability. Although a possible mosaic expression pattern of the MMTV promoter [43] cannot be discounted, differing percentages of stromal PPARγ expressing cells may also explain the variability of PPARγ expression observed in PPARγ-MG KO mammary tumours. This is supported by our IF data showing specificity and extent of PPARγ deletion among mammary epithelial cells of untreated PPARγ-MG KO mice. It is also possible that other non-mammary epithelial cell sources of PPARγ signaling may have contributed to the outcomes of these *in vivo* tumourigenesis studies. We previously showed that mammary adipocyte-specific PPARγ blocks breast tumour progression in part via upregulation of BRCA1 [13]. Interestingly, in the present study, treatment with ROSI caused induction of BRCA1 expression in PPARγ-WT, but not PPARγ-MG KO mouse mammary tumours. This suggests activation of PPARγ may protect against breast tumour progression only when mammary epithelial-stromal crosstalk contains functional PPARγ signaling in both cell types, and is the focus of additional studies beyond the scope of this work.

Untreated knockout serum contained lower levels of known proinflammatory and chemotactic cytokines, including eotaxin, IFN-γ and MCP-1α [44-46], as well as other contextually-dependent proinflammatory signals, such as IL-4 and GM-CSF [45], that could have possibly rendered PPARγ-MG KOs less susceptible to breast cancer compared to PPARγ-WTs when challenged with DMBA. DMBA + ROSI cotreatment also rescued PPARγ-WTs via downregulation of serum VEGF. This may be the result of direct PPARγ activity via a PPRE in the VEGF promoter [47], or indirectly via other PPARγ targets such as BRCA1, which can silence VEGF expression and secretion [48], or Cox-2, which can induce VEGF expression [49].

Irrespective of treatment, all PPARγ-MG KOs exhibited significantly lower levels of serum IL-4, IL-10, IL-13, eotaxin, GM-CSF, IFN-γ and MIP-1α compared to PPARγ-WT mice. This is particularly intriguing because these cytokines are commonly produced by macrophages and T lymphocytes [44,46,50,51]. Given that this cytokine expression pattern is genotype-specific raises the possibility that PPARγ-MG KO mice possess fewer macrophages and T cells, and thus experience reduced inflammation, compared to PPARγ-WTs. This explanation may provide another layer why knockout mice were less susceptible to breast tumourigenesis when challenged with DMBA.

## Conclusion

**Figure 7:**
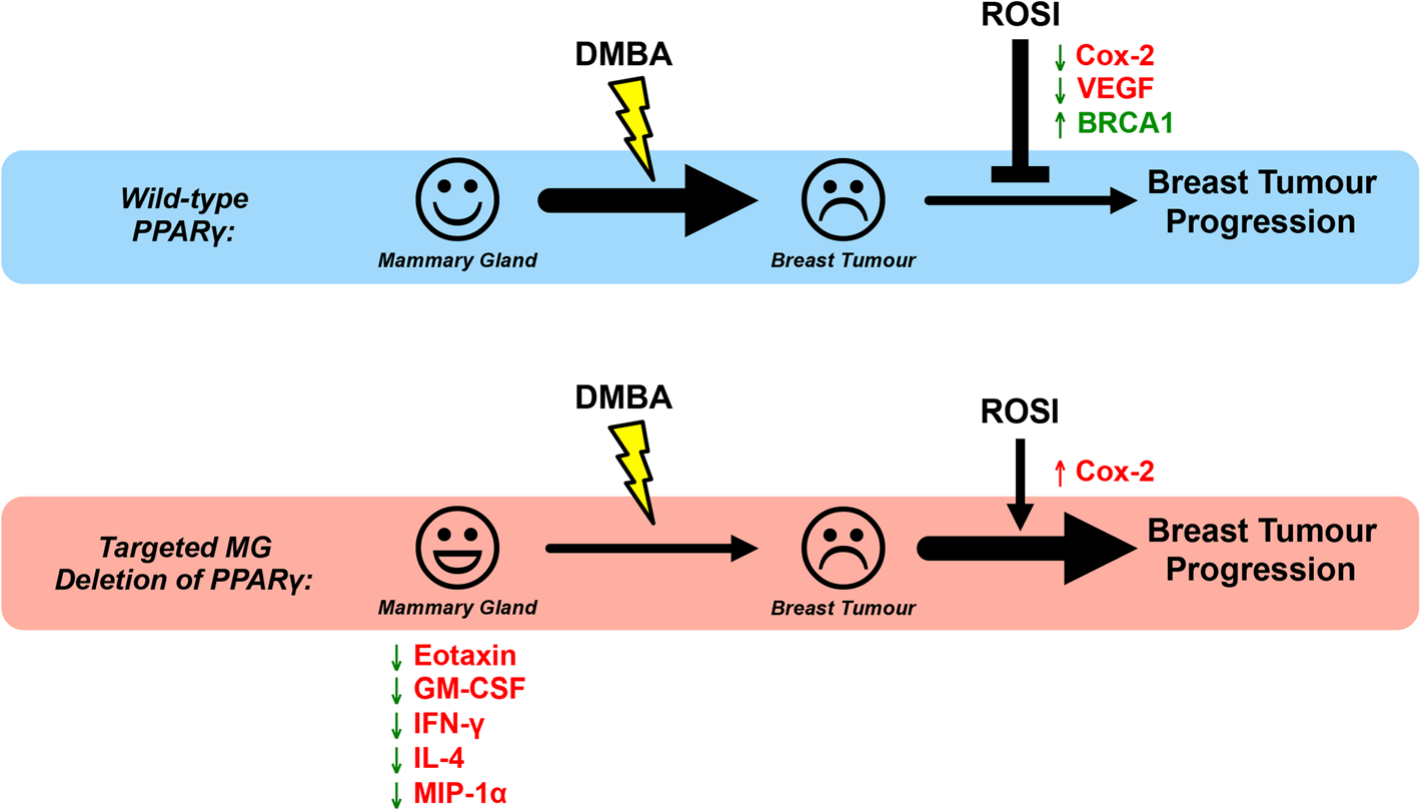
Big picture summary of the effects of MG-specific PPARγ loss. PPARγ-MG KO mice have decreased serum levels of proinflammatory and chemotactic cytokines (IL-4, eotaxin, GM-CSF, IFN-γ, and MIP-1α) which may, in part, contribute to their decreased susceptibility to DMBA Only-mediated carcinogenesis compared to PPARγ-WTs. Activation of PPARγ in MG cells suppresses breast tumourigenesis in PPARγ-WT mice by increasing BRCA1 and suppressing VEGF and Cox-2 expression, effectively rescuing PPARγ-WT mice from breast tumour progression. In MG cells lacking PPARγ expression, DMBA-induced breast tumour progression is enhanced by cotreatment with a PPARγ activator, due to PPARγ-independent activation of Cox-2.

A summary of MG-specific PPARγ loss (Figure 7) illustrates that reduced serum expression of the proinflammatory cytokines, IL-4, eotaxin, GM-CSF, IFN-γ, and MIP-1α, rendered PPARγ-MG KO mice less susceptible than PPARγ-WTs to DMBA-mediated breast tumourigenesis. Here we provide the first *in vivo* evidence that PPARγ activation in MG cells blocks breast tumour progression in PPARγ-WTs by upregulating BRCA1, and downregulating VEGF and Cox-2, expression. Finally, PPARγ-independent activation of Cox-2 enhanced breast tumourigenesis in PPARγ-MG KO mice. This study provides insight into the MG cell-specific role of PPARγ during DMBA-mediated breast tumour progression. The results suggest PPARγ signaling in MG cells may be required during early mammary tumourigenesis; however, activation of PPARγ within this cell population is protective against the growth and spread of breast tumours. In sharp contrast, when PPARγ signaling is disrupted in MG cells, the use of activating PPARγ ligands exert a deleterious PPARγ-independent effect. Together, these data emphasize the use of PPARγ ligands may be beneficial as novel chemotherapeutic agents for the treatment of a subpopulation of breast cancer patients, and that PPARγ expression may serve as a strong predictive biomarker of patient response.

## Materials and methods

### Animals

All mice were housed and treated in accordance with Canadian Council for Animal Care (CCAC) guidelines under animal protocols approved by the Queen’s University Animal Care Committee (UACC) as previously described [14]. Transgenic mice expressing the MMTV-LTR-Cre^+^ gene were obtained from the NCI-Frederick repository (Frederick, Maryland), and crossed with our previously generated PPARγ^(fl/fl)^;Cre^−^ (PPARγ-WT) mice [20], to produce PPARγ^(fl/fl)^;MMTV-LTR-Cre^+^ (PPARγ-MG KO) mice. Mouse genotypes were confirmed by PCR analysis (Additional file 2: Figure S2) as before [12].

### In vivo breast tumourigenesis

At age 8-12 weeks, PPARγ-WT and PPARγ-MG KO virgin female mice received 1 mg DMBA (Sigma-Aldrich, D3254) by gavage once/week for 6 weeks. At week 7, randomized mice either continued on a regular chow diet (DMBA Only: PPARγ-WT, n = 25 and PPARγ-MG KO, n = 39) or received a PPARγ ligand (ROSI; 4 mg/kg/day)-supplemented chow diet (DMBA + ROSI: PPARγ-WT, n = 34 and PPARγ-MG KO, n = 17) for the study duration. Mice were monitored for tumourigenic changes for 25 weeks, and tumour samples were harvested as previously described [13]. Non-fasted submandibular blood was obtained pre-, mid- and end-study, and separated to obtain serum samples that were frozen in liquid N_2_ for future analysis. Pathological staging of tumours was performed in a blinded fashion by collaborating pathologists.

### Immunofluorescent (IF) staining

Formalin-fixed paraffin-embedded untreated mammary glands and mammary tumours from PPARγ-WT and PPARγ-MG KOs in each treatment group were sectioned and stained as described previously [14]. Sections were stained with primary antibodies for pan-cytokeratin (Dako, M3515; 1:500 dilution) and PPARγ (Santa Cruz, sc-7196; 1:500 dilution) or BRCA1 (Santa Cruz, sc-7867; 1:500 dilution). Secondary antibodies used were donkey α-rabbit FITC (Santa Cruz, sc-2090; 1:500 dilution) and α-mouse Alexa Fluor 594 (Invitrogen, A11005; 1:500 dilution). Slides were coverslipped with mounting media containing DAPI stain (Vectashield). IF staining was visualized with a BX51 System Microscope (Olympus). Images were acquired with QCapture Pro 5.1 software (QImaging) and analyzed with Image-Pro Plus 6.0 software (Media Cybernetics).

### Immunoblotting

Whole-cell extracts were prepared from normal and tumour tissue samples from PPARγ-WT and PPARγ-MG KO mice as previously described [14]. Protein concentrations were quantified using the *DC* protein assay (BioRad). Proteins were detected with primary antibodies for PPARγ (Santa Cruz, sc-7273; 1:500 dilution), β-actin (Santa Cruz, sc-47778; 1:000 dilution), Cox-2 (Cayman Chemical, #160126; 1:500 dilution) and PTEN (Cell Signaling, #9559; 1:1,000 dilution) followed by appropriate HRP-conjugated secondary goat α-mouse (Santa Cruz, sc-2005; 1:10,000 dilution) or goat α-rabbit (Santa Cruz, sc-2004; 1:10,000 dilution) antibodies. Protein expression was assessed using ImageJ analysis software (rsbweb.NIH.gov).

### Serum assays

A Bio-Plex Pro Mouse Cytokine 23-plex serum assay kit (BioRad Laboratories) was used to assess cytokine concentrations of IL-1α, IL-1β, IL-2, IL-3, IL-4, IL-5, IL-6, IL-9, IL-10, IL-12(p40), IL-12(p70), IL-13, IL-17A, eotaxin, G-CSF, GM-CSF, IFN-γ, KC, MCP-1, MIP-1α, MIP-1β, RANTES, and TNF-α as previously described [14]. Clustering and heat map analyses were performed with Cluster 3.0 and TreeView software (Stanford University). Serum VEGF, leptin and prostaglandin E (PGE) metabolites were analyzed using ELISA kits as per manufacturer’s (Cayman Chemical) instructions. All cytokine concentrations are reported as the mean ± standard deviation (SD) pg/ml.

### Statistical analysis

Differences between genotype and treatment groups were assessed using a Two-Way analysis of variance (ANOVA), followed by a Tukey’s post-hoc test for group comparisons. Survival was analyzed using a Log Rank test, and proportions were assessed using Chi-square analysis. GraphPad Prism (Version 6.0) software was used for all analyses. A value of p < 0.05 was considered statistically significant.

## Additional files

Additional file 1: Figure S1. PPARγ and BRCA1 expression in untreated tissue. Representative immunofluorescence images illustrating expression of cell nuclei (DAPI; in blue), PPARγ or BRCA1 (in green) and cytokeratin (CK; in red), with an accompanying composite image, in untreated virgin mammary tissue from PPARγ-WT and PPARγ-MG KO mice. All photos taken at × 600. Scale bar, 50 μm. Additional file 2: Figure S2. Mouse genotyping of PPARγ. Mice were genotyped using a standard polymerase chain reaction (PCR) assay as previously described [12]. Representative PCR results obtained using DNA isolated from tails of (n = 3) PPARγ-WT and PPARγ-MG KO mice. Floxed PPARγ allele, ~285 bp; *Cre*-mediated recombined null allele, ~450 bp.
