# Supplementary figures and images for: Opposing roles for mammary epithelial-specific PPARγ signaling and activation during breast tumour progression
Source: Mol Cancer. 2015 Apr 15;14:85. doi: 10.1186/s12943-015-0347-8 (PMC4422298; doi:10.1186/s12943-015-0347-8)

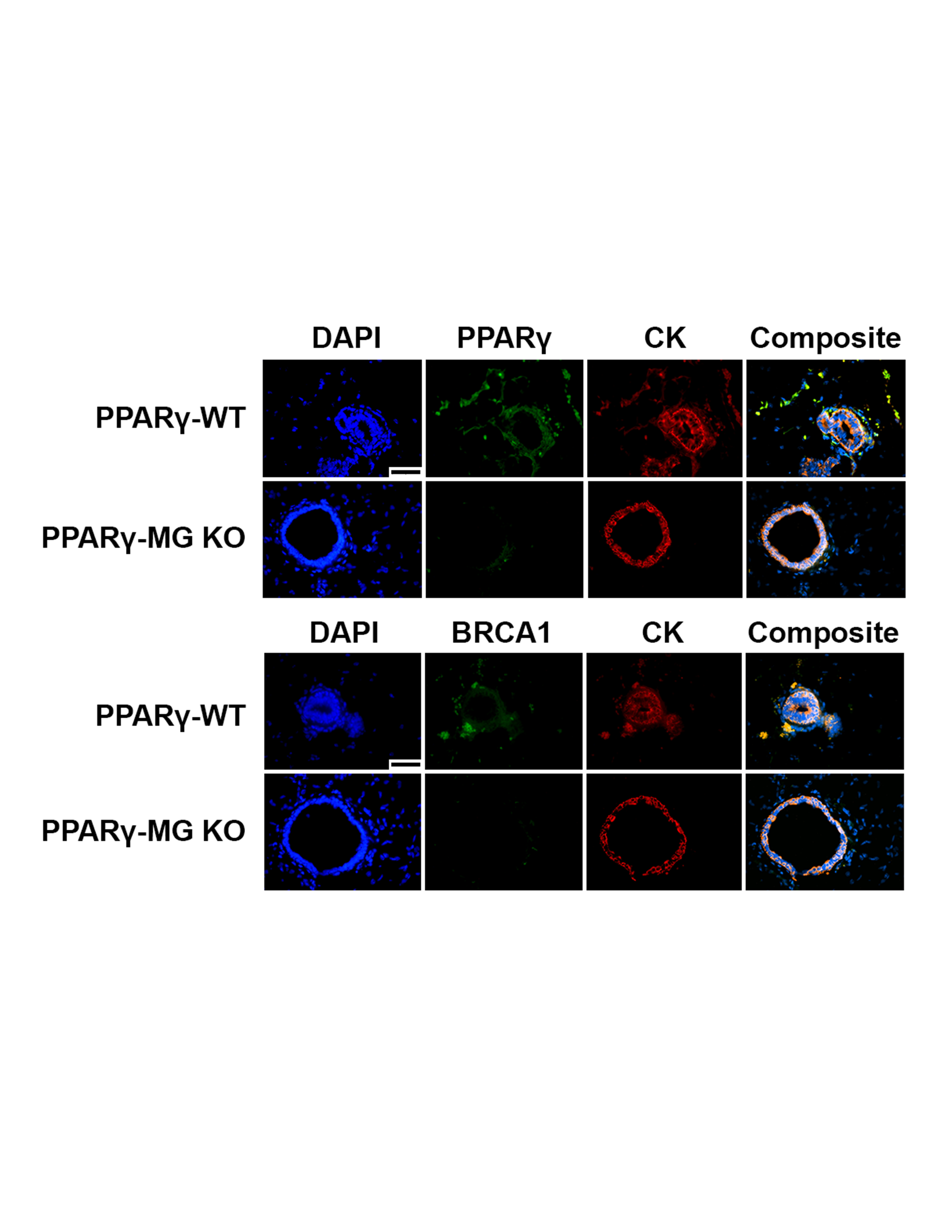

Supplement: Additional file 1: Figure S1. — PPARγ and BRCA1 expression in untreated tissue. Representative immunofluorescence images illustrating expression of cell nuclei (DAPI; in blue), PPARγ or BRCA1 (in green) and cytokeratin (CK; in red), with an accompanying composite image, in untreated virgin mammary tissue from PPARγ-WT and PPARγ-MG KO mice. All photos taken at × 600. Scale bar, 50 μm. [file 12943_2015_347_MOESM1_ESM.png]

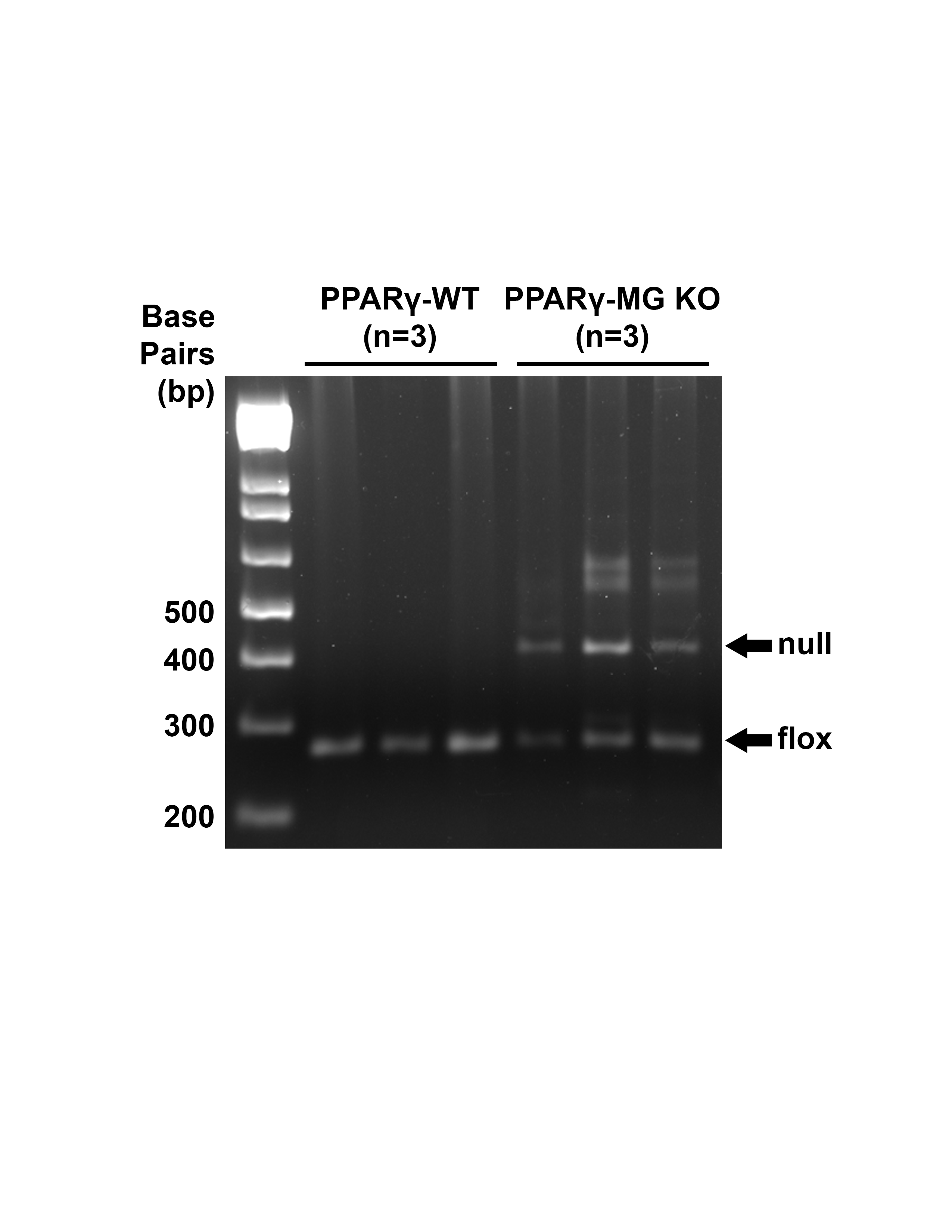

Supplement: Additional file 2: Figure S2. — Mouse genotyping of PPARγ. Mice were genotyped using a standard polymerase chain reaction (PCR) assay as previously described [12]. Representative PCR results obtained using DNA isolated from tails of (n = 3) PPARγ-WT and PPARγ-MG KO mice. Floxed PPARγ allele, ~285 bp; Cre-mediated recombined null allele, ~450 bp. [file 12943_2015_347_MOESM2_ESM.png]
